# Visceral to subcutaneous fat area ratio as a novel prognostic biomarker in cirrhosis patients undergoing TIPS: a retrospective study

**DOI:** 10.3389/fmed.2026.1769708

**Published:** 2026-03-19

**Authors:** Xiangrui Li, Qin Yin, Jiangqiang Xiao, Xiaotian Chen, Ming Zhang, Bo Gao

**Affiliations:** 1 Department of Clinical Nutrition, Nanjing Drum Tower Hospital, Affiliated Hospital of Medical School, Nanjing University, Nanjing, China; 2 Department of Gastroenterology, Nanjing Drum Tower Hospital, Affiliated Hospital of Medical School, Nanjing University, Nanjing, China; 3 Department of Gastroenterology, Beijing Ditan Hospital, Capital Medical University, Beijing, China

**Keywords:** hepatic encephalopathy, prognostic biomarker, subcutaneous fat area, TIPS, visceral fat area

## Abstract

**Aims:**

This research aims to explore the predictive value of the visceral-to-subcutaneous fat area ratio (V/S ratio) for post-TIPS overt hepatic encephalopathy (OHE) and transplant-free survival (TFS).

**Methods:**

A total of 239 cirrhosis patients were included. Receiver operating characteristic (ROC) curves were generated to identify cutoff points for both visceral fat area (VFA) and VFA to subcutaneous fat area (SFA) ratio (V/S ratio) as predictors of 1-year OHE and 2-year TFS. Kaplan–Meier and Cox proportional hazards modeling to determine associations between body composition characteristics and 1-year OHE and 2-year TFS.

**Results:**

ROC analysis and AUROC demonstrated that V/S ratio and VFA had significant and satisfactory prediction ability for 1-year OHE at a level of 1.041 (AUROC 0.817, *p* < 0.001) and 87.12cm^2^ (AUROC 0.705, *p* < 0.001), respectively. In the Cox multivariate regression model, age, V/S ratio, creatinine, and combined sarcopenia and myosteatosis were risk factors for 1-year OHE. High V/S ratio was associated with a 1.75-fold overall risk of 1-year OHE (HR 1.748, 95% CI 1.438–2.124, *p* < 0.001) and a 1.55-fold overall risk of death/liver transplantation (HR 1.547, 95% CI 1.199–1.997, *p* = 0.001) compared to patients with a low V/S ratio.

**Conclusion:**

Preoperative assessment of the V/S ratio is beneficial for predicting OHE and TFS after TIPS.

## 1 Introduction

Liver cirrhosis is the end-stage manifestation of chronic liver disease. It carries a risk of mortality, which is associated with the development of decompensating events related to portal hypertension, such as variceal bleeding and refractory ascites (1). A transjugular intrahepatic portosystemic shunt (TIPS) reduces portal vein pressure by establishing an intrahepatic shunt between the portal vein and the hepatic vein, and it is a critical intervention for managing complications of portal hypertension in cirrhosis (2). TIPS not only demonstrates significant efficacy in treating complications of portal hypertension, but a meta-analysis also suggested that it could improve patients’ nutritional status, as evidenced by increases in ascites-free body weight, body mass index (BMI), and muscle mass (3–5). However, postoperative prognosis remains variable among patients. Adverse outcomes such as hepatic encephalopathy (HE) and death are influenced by multiple factors (6). The incidence of short-term HE after TIPS is higher than before the operation (7). Although it remains controversial, some studies suggest that HE may affect quality of life and survival (8, 9).

Traditional prognostic models, such as the model for end-stage liver disease (MELD) and Child-Turcotte-Pugh (CTP) scores, focus on liver function and the severity of ascites but do not account for metabolic and inflammatory risks, which may limit their predictive accuracy in patients undergoing TIPS (10). Although MELD was initially designed to predict survival after TIPS surgery, it has a poor correlation with the occurrence of HE, which is influenced by factors such as ammonia metabolism (11). Similarly, the CTP score accounts for ascites severity. However, the assessment of ascites is influenced by subjective factors (12).

A recent study emphasized the prognostic value of body composition analysis in liver cirrhosis (13). Compared with traditional anthropometric indicators, such as BMI, visceral adipose tissue (VAT) has become a superior predictor of clinical outcomes (14). VAT plays a key role in the pathophysiology of metabolic dysfunction-associated steatotic liver disease and its advancement to cirrhosis (15). VAT is not only an energy storage organ but also an active endocrine organ that secretes pro-inflammatory cytokines and adipokines, exacerbating insulin resistance, systemic inflammation, and liver fibrosis (16). The accumulation of VAT is associated with accelerated liver decompensation and impaired regenerative capacity, which may deteriorate the outcome of cirrhotic patients (17). In contrast, one study found that subcutaneous fat may play a protective role in female patients with cirrhosis (18). A study from United States found that visceral adipose tissue inflammation is highly prevalent in patients with cirrhosis, and an elevated visceral-to-subcutaneous adipose tissue ratio can independently predict visceral adipose tissue inflammation, serving as a prognostic imaging marker for adverse outcomes in liver transplant recipients (19). However, a recent study found that lower subcutaneous fat area (SFA) and visceral fat area (VFA) were significantly associated with 90-day mortality, whereas the visceral-to-subcutaneous fat ratio (V/S ratio) showed no significant correlation (20). Therefore, the roles of VAT and subcutaneous fat in predicting TIPS outcomes remain incompletely understood. Furthermore, the cutoff value for the occurrence of HE in cirrhotic patients who have visceral obesity has not yet been determined. Since, the predictive value of visceral fat and fat distribution for TIPS prognosis warrants exploration.

The objective of this study was to assess the V/S ratio as a predictive biomarker for post-TIPS complications, including HE and transplant-free survival (TFS). By integrating these body composition indicators, the study aims to identify novel therapeutic targets for optimizing post-TIPS outcomes in cirrhotic patients.

## 2 Methods

### 2.1 Participants

Liver cirrhotic patients who were hospitalized in the gastroenterology department of Nanjing Drum Tower Hospital from January 2017 to December 2020 were retrospectively collected. The Clinical Research Ethics Committee of Nanjing Drum Tower Hospital approved this study. Before the TIPS procedure, written informed consent was acquired from each participant. Inclusion criteria were: (1) Aged over 18; (2) Clinically and radiographically diagnosed cirrhosis; (3) Received TIPS for the first time; (4) Abdominal CT examination before treatment; (5) Regular follow-up for at least 1 year. Patients were ineligible if: (1) Underwent any previous liver-related surgical procedure; (2) Malignant tumor; (3) Severe heart, lung, and kidney dysfunction; (4) Mental disorders or use of psychotropic drugs; (5) Pregnancy and breastfeeding period; (6) TIPS for urgent settings; (7) Unsuccessful TIPS; (8) Incomplete medical record; (9) Loss of follow-up.

### 2.2 Data collection

Baseline data for patients before TIPS were collected, including gender, age, weight, height, etiology of cirrhosis (viral, alcoholic, or other), diabetes, splenectomy history, presence of ascites, and previous history of bleeding or HE. Ascites was graded according to the 2023 Chinese guidelines on the management of ascites in cirrhosis by the Chinese Society of Hepatology, Chinese Medical Association as follows: mild (Grade 1, only detectable by ultrasound, ascites depth < 3 cm under ultrasonography), moderate (Grade 2, moderate symmetric distension of the abdomen, ascites depth is 3–10 cm under ultrasonography), and severe (Grade 3, marked distension of the abdomen, ascites depth > 10 cm under ultrasonography) (21). Each patient underwent blood tests, including serum biochemical markers, before the treatment. MELD and CTP scores were calculated by using clinical and biochemical indicators.

### 2.3 CT measurements

All patients underwent abdominal CT scans a median of 5 days (IQR: 2.8, 7.3 days) before the TIPS procedure. Skeletal muscle mass, skeletal muscle attenuation, SFA, and VFA were quantified independently by two professional radiologists at the level of the L3 on the CT image. In cases where discrepancies occurred between the two radiologists, the mean value of the two measurements was calculated and used for analysis. The body composition assessment method was adopted as previously described (22). Body composition measurements were analyzed using MATLAB software (MathWorks, Massachusetts, United States). Different body composition tissue compartments were manually outlined based on specific Hounsfield unit (HU) thresholds: skeletal muscle (−29 to 150 HU), visceral fat (−150 to −50 HU), and subcutaneous fat (−190 to −30 HU). Total areas for each compartment were subsequently calculated automatically. Skeletal muscle index (SMI) and a fat distribution indicator (V/S ratio) were then calculated. Based on a multicenter study of Chinese individuals, the diagnostic cut-off values for sarcopenia were defined as L3-SMI < 44.77 cm^2^/m^2^ for males and <32.50 cm^2^/m^2^ for females (23). According to a previous study from Cola et al., myosteatosis was defined as skeletal muscle attenuation at L3 < 41 HU for patients with BMI < 25 kg/m^2^ and <33HU for patients with BMI ≥ 25 kg/m^2^, respectively (24).

### 2.4 TIPS procedure

The catheter sheath was sent into the inferior vena cava through the right internal jugular vein. Following access of the right femoral artery, indirect portal venography was performed through either the superior mesenteric artery or splenic artery to display the main left and right branches of the portal vein. Subsequently, the puncture was performed from the hepatic vein to the intrahepatic portal vein branch, and the catheter sheath was positioned within the central portal vein for pressure measurement and direct portal venography. A covered PTFE stent (Bard, United States or Gore, United States), or a covered PTFE stent combined with a bare-metal stent (Bard, United States) was inserted along the guide wire into the appropriate position in the liver and expanded with the balloon to the same diameter. Immediately after stent placement, the postoperative portal vein pressure was re-measured.

### 2.5 Postoperative follow-up

All patients received regular follow-up after discharge at 1, 3, 6, and 12 months, once a year thereafter. Postoperative history, laboratory data and abdominal ultrasound were collected during follow-up. The primary endpoint of the study was TFS, defined as the time from enrollment to either death from any cause or liver transplantation, whichever occurred first. The secondary endpoint was 1-year overt HE (OHE). At every post-TIPS clinical visit, signs and symptoms of HE were graded using the West Haven by a qualified hepatologist (25). OHE was defined as West Haven grade ≥2 (grades 2, 3, or 4), characterized by the presence of mental abnormalities (including personality and behavior changes) and neurological abnormalities (such as coma). Once a patient experienced such an event, they were considered to have reached the secondary endpoint.

### 2.6 Statistical analysis

Data were analyzed by SPSS, version 26.0 (IBM, New York, United States). The continuous variables were expressed as mean ± SD or median (25th percentile and 75th percentile). Continuous data were evaluated using the t-test or the Mann–Whitney U-test. All categorical variables were presented as percentages and statistically assessed using χ^2^ tests for both VFA and V/S ratio. Cutoff values were identified using Receiver operating characteristic (ROC) curves to predict the prognosis of cirrhotic patients after TIPS. The optimal cutoff point was defined by using the Youden Index (sensitivity + specificity – 1). The Delong test was used to evaluate differences in the areas under the curves (AUCs). The Spearman correlation test investigated relationships between DM and body measurement indicators. Kaplan–Meier analysis was used to analyze the relationships between V/S ratio and incidence of 1-year OHE and 2-year death or liver transplantation. Cox proportional hazards regression was conducted in two stages (univariable and multivariable) to identify risk factors for mortality and OHE. Hazard rates with 95% confidence intervals (CIs) were calculated. A two-sided *p* value < 0.05 was considered statistically significant.

## 3 Results

### 3.1 Baseline characteristics

In total, 239 patients with liver cirrhosis were finally included in this study (Supplementary Figure 1). Demographic and clinical features at baseline are shown in Table 1. The cohort comprised 139 males (58.2%) and 100 females (42.8%) with a mean age of 57.72 ± 11.40 years. The overall the rate of shunt dysfunction was 8.4%, the rebleeding rate was 10.5%. Furthermore, a total of 41 deaths occurred during the 2-year follow-up period. The detailed causes of death are as follows: 23 patients (56.1%) died from liver failure and hepatic encephalopathy, 5(12.2%) patients died from infection, 6(14.6%) patients died from variceal rebleeding, and 7(17.1%) patients died from other causes (such as cerebral hemorrhage, acute renal failure).

**Table 1:** Baseline characteristics.

Variable	All patients	High V/S (*n* = 118)	Low V/S (*n* = 121)	*p*
Age (Year)	57.72 ± 11.40	57.58 ± 12.10	57.85 ± 10.72	0.853
Gender (Male/Female)	139/100(58.2/41.8%)	83/35(70.3/29.7%)	56/65(46.3/53.7%)	<0.001***
Body height(cm)	165.57 ± 7.20	166.63 ± 7.30	164.55 ± 6.97	0.025*
Body weight(kg)	60.59 ± 10.46	59.49 ± 9.75	61.66 ± 11.04	0.109
BMI (kg/m2)	22.04 ± 3.13	21.37 ± 2.80	22.69 ± 3.30	0.001**
Etiology (virus/alcoholic/others; n, %)	121/24/94 (40.6/8.1/31.5%)	63/13/42 (53.4/11.0/35.6%)	58/11/52 (47.9/9.1/43.0%)	0.497
Previous history of bleeding (none/1/2/3 and more; n, %)	21/62/34/122 (8.8/25.9/14.2/51.0%)	11/30/15/62 (9.3/25.4/12.7/52.5%)	10/32/19/60 (8.3/26.4/15.7/49.6%)	0.901
Previous history of HE (n, %)	4(1.7%)	1(0.8%)	3(2.5%)	0.325
CTP score	7.10 ± 1.24	7.17 ± 1.15	7.02 ± 1.31	0.366
CTP class (A/B/C)	77/158/4 (32.2/66.1/1.7%)	35/81/2 (29.7/68.6/1.7%)	42/77/2 (34.7/63.6/1.7%)	0.705
MELD score	10.08 ± 2.66	10.23 ± 2.85	9.94 ± 2.47	0.406
Skeletal muscle mass (cm^2^)	117.83 ± 29.30	114.48 ± 27.70	121.10 ± 30.54	0.080
Skeletal muscle attenuation (HU)	35.52 ± 9.41	35.54 ± 9.57	3.50 ± 9.29	0.980
SMI (cm^2^/m^2^)	42.76 ± 9.31	41.03 ± 8.73	44.44 ± 9.57	0.004**
Sarcopenia (n, %)	86(36.0%)	61(51.7%)	25(20.7%)	<0.001***
Myosteatosis (n, %)	151(63.2%)	74(62.7%)	77(63.6%)	0.882
Sarco-myo (n, %)	59(24.7%)	40(33.9%)	19(15.7%)	0.001**
SFA (cm^2^)	88.82 ± 49.24	64.45 ± 33.74	113.55 ± 49.48	<0.001***
VFA (cm^2^)	89.13 ± 40.30	99.18 ± 42.18	79.33 ± 35.91	<0.001***
V/S ratio	1.26 ± 0.82	1.81 ± 0.85	0.72 ± 0.20	<0.001***
Ascites (None/Mild/Moderate/ Severe; n, %)	54/49/99/37 (22.6/20.5/41.4/15.5%)	21/24/56/17 (17.8/20.3/47.5/14.4%)	33/25/43/20 (27.3/20.7/35.5/16.5%)	0.204
Laboratory				
ALT (U/L)	19.8 (13.7, 26.8)	18.2(13.3,26.6)	21.5(14.1,28.0)	0.148
AST(U/L)	27.2 (20.6, 37.9)	25.8(18.4,37.1)	27.5(22.4,38.7)	0.131
TBIL (umol/L)	16.7 (12.0, 23.2)	17.5(12.4,22.9)	16.1(11.9,23.9)	0.482
Albumin(g/L)	33.09 ± 4.02	33.47 ± 4.12	32.70 ± 3.88	0.139
Creatinine(umol/L)	63.0(51.0,73.0)	60.0(51.8,79.0)	64.0(51.0,69.0)	0.044*
WBC (10^9/L)	2.8 (1.9, 5.0)	2.9(2.0,4.9)	2.6(1.9,5.3)	0.852
Platelet(10^9/L)	65.0 (43.0, 112.0)	61.5(44.5,141.5)	68.0(42.5,107.5)	0.778
PT(s)	14.40 ± 1.97	14.42 ± 2.02	14.37 ± 1.92	0.832
INR	1.25 (1.14, 1.36)	1.26(1.15,1.37)	1.24(1.14,1.37)	0.624
Fibrinogen(g/L)	1.6(1.2,2.1)	1.6(1.3,2.2)	1.6(1.2,2.1)	0.171
Diabetes (n, %)	51(21.3%)	26(22.0%)	25(20.7%)	0.796
Splenectomy history (n, %)	48(20.1%)	27(22.9%)	21(17.4%)	0.286
Indication for TIPS (Gastrointestinal bleeding/ Portal vein thrombosis/ Refractory ascites/ Others; n, %)	213/10/13/3 (89.1/4.2/5.4/1.3%)	104/5/9/0 (88.1/4.2/7.6/0%)	109/5/4/3 (90.1/4.1/3.3/2.5%)	0.172
TIPS procedure				
Pre-TIPS RAP (mmHg)	5.22 ± 2.88	4.91 ± 2.76	5.52 ± 2.98	0.100
Pre-TIPS IVCP (mmHg)	6.94 ± 3.14	6.81 ± 3.13	7.07 ± 3.16	0.522
Pre-TIPS PVP (mmHg)	30.82 ± 5.94	30.94 ± 5.91	30.70 ± 5.99	0.749
Post-TIPS RAP (mmHg)	10.00 ± 3.80	10.15 ± 3.44	9.84 ± 4.13	0.530
Post-TIPS IVCP (mmHg)	10.22 ± 3.75	10.35 ± 3.30	10.09 ± 4.16	0.598
Post-TIPS PVP (mmHg)	19.94 ± 5.46	19.94 ± 4.93	19.93 ± 5.95	0.978
OHE after TIPS within 1 year (n, %)	50(20.9%)	45(38.1%)	5(4.1%)	<0.001***
Grade of OHE (2/3/4)	47/2/1 (94.0/4.0/2.0%)	42/2/1 (93.3/4.5/2.2%)	5/0/0 (100/0/0%)	0.838
Shunt dysfunction after TIPS within 1 year (n, %)	20(8.4%)	14(11.9%)	6(5.0%)	0.054
Rebleeding after TIPS within 1 year	25(10.5%)	18(15.3%)	7(5.8%)	0.017*
Deaths/liver transplantation after TIPS within 2 years (n, %)	44(18.4%)	30(25.4%)	14(11.6%)	0.006**

* indicated for *p* < 0.05; ** indicated for *p* < 0.01; *** indicated for *p* < 0.001. BMI, body mass index; OHE, overt hepatic encephalopathy; CTP, Child-Turcotte-Pugh; MELD, Model for end-stage liver disease; SMI, skeletal muscle index; Sarco-myo, combined sarcopenia and myosteatosis; SFA, subcutaneous fat area; VFA, visceral fat area; V/S ratio, subcutaneous fat area to visceral fat area ratio; ALT, alanine aminotransferase; AST, aspartate aminotransferase; TBIL, total bilirubin; WBC, white blood cell; PT, prothrombin time; INR, international normalized ratio; TIPS, transjugular intrahepatic portosystemic shunt; RAP, right atrial pressure; IVCP, inferior vena cava pressure; PVP, portal venous pressure.

### 3.2 Cut-off values for VFA to SFA ratio

**Figure 1:**
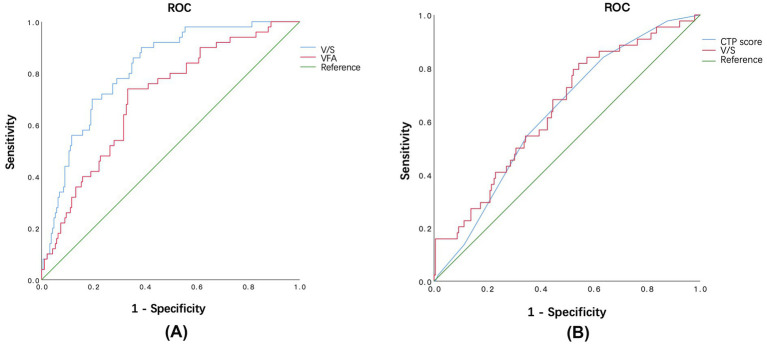
**(A)** The ROC curves of V/S and VFA for the prediction of 1-year OHE (V/S ratio: AUROC = 0.817 ± 0.030, *p* < 0.001, 95%CI: 0.7570.877. The optimal cut-off value of V/S in prediction of 1-year OHE was 1.041. VFA: AUROC = 0.705 ± 0.040, *p* < 0.001, 95% CI: 0.6270.783. The optimal cut-off value of VFA in prediction of 1-year OHE was 87.12cm^2^ in overall patients enrolled.). **(B)** The ROC curves of V/S and CTP score for the prediction of 2-year death or liver transplantation (V/S ratio: AUROC = 0.645 ± 0.045, *p* = 0.003, 95% CI: 0.558–0.733. The optimal cut-off value of V/S in prediction of 2-year death or liver transplantation was 0.904. CTP score: AUROC = 0.628 ± 0.043, *p* = 0.008, 95% CI: 0.544–0.712. The optimal cut-off value of VFA in prediction of 2-year death or liver transplantation was 6.5 in overall patients enrolled.).

ROC analysis demonstrated that V/S ratio and VFA had significant and satisfactory predict capacity for 1-year OHE, with optimal cut-offs of 1.041 (AUROC 0.817 ± 0.030, *p* < 0.001, 95% CI: 0.757–0.877) and 87.12cm^2^ (0. 705 ± 0.040, *p* < 0.001, 95%CI: 0.627–0.783), respectively (Figure 1A; Table 2). Moreover, the V/S ratio showed superior predictive performance for OHE compared with VFA, as assessed by the DeLong test (*P* for Z = 0.046). For mortality or liver transplantation prediction after TIPS procedures, V/S ratio and CTP score showed significant predictive advantages, with cutoff values of 0.904 (*p* = 0.003) and 6.5 (*p* = 0.008), respectively (Figure 1B; Table 2).

**Table 2:** ROC of Visceral fat area and Subcutaneous fat area ratio in predicting prognosis of cirrhosis patients after TIPS.

Variable	OHE within 1 year							Death or liver transplantation within 2 years					
	Cut-off	AUC	Sensitivity	Specificity	Youden index	*p*	*P* for Z	Cut-off	AUC	Sensitivity	Specificity	Youden index	*p*
V/S ratio	1.041	0.817	0.900	0.614	0.514	<0.001***		0.904	0.645	0.818	0.456	0.274	0.003**
VFA	87.123	0.705	0.740	0.667	0.407	<0.001***	0.046*	98.107	0.557	0.455	0.703	0.158	0.237
CTP score	7.5	0.579	0.480	0.640	0.120	0.084	<0.001***	6.5	0.628	0.841	0.364	0.205	0.008**
MELD score	12.5	0.547	0.280	0.862	0.142	0.311	<0.001***	13.5	0.578	0.205	0.928	0.133	0.104

* indicated for *p* < 0.05; ** indicated for *p* < 0.01; *** indicated for *p* < 0.001. OHE, overt hepatic encephalopathy; CTP, Child-Turcotte-Pugh; MELD, Model for end-stage liver disease; VFA, visceral fat area; V/S ratio, subcutaneous fat area to visceral fat area ratio.

Patients were categorized into high (*n* = 118) and low V/S ratio groups (*n* = 121) according to the predetermined cutoff point (Table 1). Participants’ gender, height, and BMI were significantly different between the two groups (*p* < 0.05). The high V/S group had significantly lower BMI, SMI, and a higher prevalence of sarcopenia (all *p* < 0.001). Notably, the cumulative 1-year incidence of OHE and the 2-year incidence of death or liver transplantation were also markedly higher in the high V/S population than in the low V/S population (*p* < 0.001 and *p* = 0.006, respectively).

### 3.3 Associations of diabetes with body composition

When exploring the relationship between diabetes and body composition profiles, VFA showed a significant correlation with diabetes (*p* = 0.027), whereas BMI and other muscle-related indicators showed no significant association (Table 3).

**Table 3:** Relationship between diabetes and body measurement indicators.

Variable	r	*p*
BMI	0.110	0.090
Skeletal muscle mass	0.012	0.852
SMI	0.024	0.717
Sarcopenia	−0.050	0.441
SFA	0.122	0.060
VFA	0.143	0.027*
Myosteatosis	0.059	0.365
Sarco-myo	0.010	0.881

* indicated for *p* < 0.05; ** indicated for *p* < 0.01; *** indicated for *p* < 0.001. BMI, body mass index; SMI, skeletal muscle index; SFA, subcutaneous fat area; VFA, visceral fat area; Sarco-myo, combined sarcopenia and myosteatosis.

### 3.4 Cox regression of body composition profiles with OHE and death/liver transplantation after TIPS

**Figure 2:**
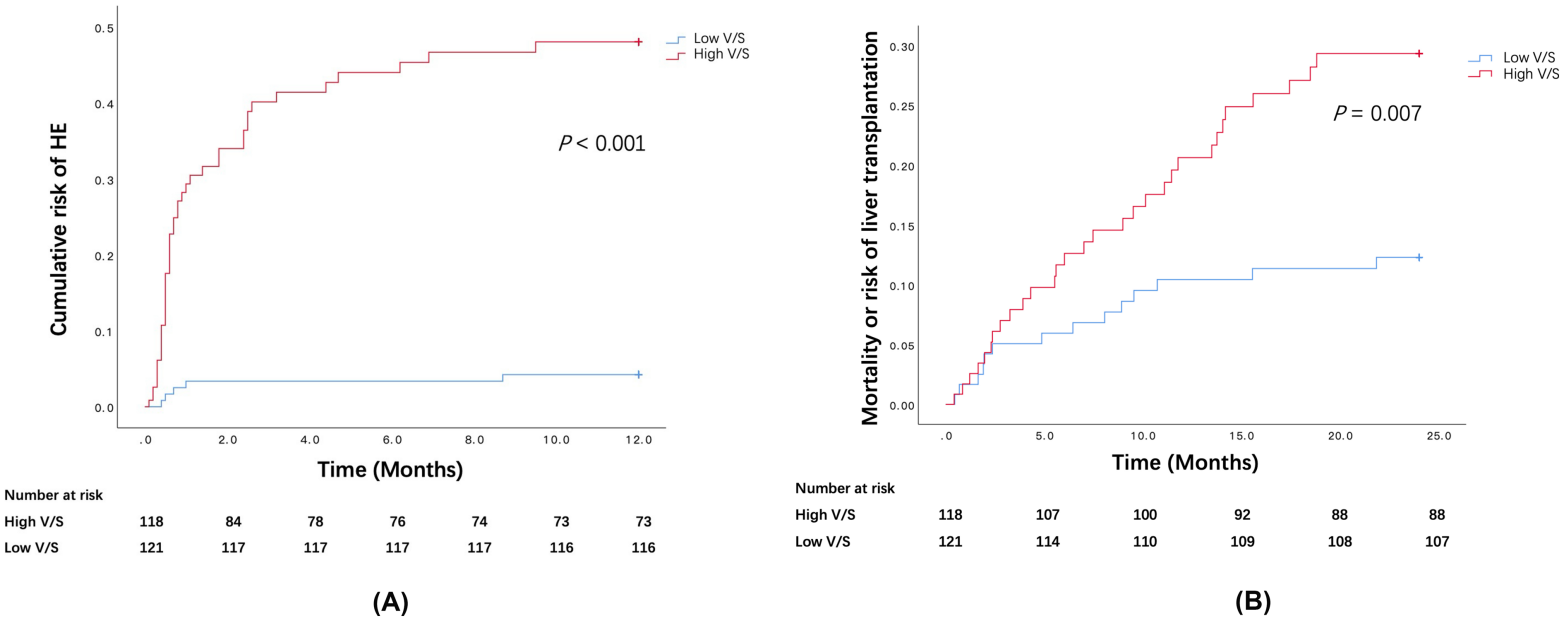
**(A)** Cumulative risk of 1-year OHE in patients with low V/S and high V/S after TIPS. **(B)** Mortality or risk of liver transplantation within 2 years in patients with low V/S and high V/S after TIPS.

Figures 2A,B shows the cumulative risk of 1-year OHE and mortality or liver transplantation within 2 years in patients with low V/S and high V/S.

Univariable analysis revealed that V/S ratio (HR 1.770, 95% CI 1.487–2.107, *p* < 0.001), age (HR 1.047, 95% CI 1.021–1.073 *p* < 0.001), diabetes (HR 1.827, 95% CI 1.008–3.310 *p* = 0.047), combined sarcopenia and myosteatosis (HR 2.514, 95% CI 1.433–4.409, *p* = 0.001),creatinine(HR 1.010, 95% CI 1.005–1.016, *p* < 0.001) were significantly associated with the occurrence of 1-year OHE (Table 4). The multivariate regression model confirmed the predictive value of age, diabetes, V/S ratio, creatinine, and combined sarcopenia and myosteatosis. Moreover, patients with a high V/S ratio had a 1.75 times greater risk of developing OHE within 1 year compared to those with a low V/S ratio (HR 1.748, 95% CI 1.438–2.124, *p* < 0.001).

**Table 4:** Cox regression analysis for factors associated with 1-year OHE and 2-year death or liver transplantation.

Variable	1-year OHE						2-year death or liver transplantation					
	Univariable analysis			Multivariate analysis			Univariable analysis			Multivariate analysis		
	B	HR	*p*	B	HR	*p*	B	HR	*p*	B	HR	*p*
Age	0.046	1.047(1.021,1.073)	<0.001***	0.029	1.029(1.004,1.055)	0.023*	0.046	1.047(1.1018,1.076)	<0.001***	0.038	1.039(1.011,1.068)	0.006**
MELD score	0.084	1.088(0.988,1.198)	0.088				0.092	1.096(0.993,1.211)	0.070			
CTP score	0.198	1.219(0.980,1.517)	0.075				0.286	1.331(1.066,1.662)	0.012*	0.274	1.316(1.025,1.688)	0.031*
Diabetes	0.602	1.827(1.008,3.310)	0.047*	0.671	1.956(1.019,3.753)	0.044*	0.584	1.793(0.951,3.382)	0.071			
Sarcopenia	0.753	2.123(1.218,3.690)	0.008**				0.537	1.711(0.947,3.091)	0.075			
V/S ratio	0.571	1.770(1.487,2.107)	<0.001***	0.558	1.748(1.438,2.124)	<0.001***	0.528	1.695(1.330,2.160)	<0.001***	0.437	1.547(1.199,1.997)	<0.001***
Myosteatosis	0.559	1.749(0.930,3.291)	0.083				0.397	1.488(0.778,2.843)	0.229			
Sarco-myo	0.922	2.514(1.433,4.409)	0.001**	0.712	2.038(1.158,3.589)	0.014*	0.549	1.732(0.928,3.230)	0.084			
Previous HE	1.177	3.246(0.788,13.374)	0.103				3.028	0.048(0.000, 876.614)	0.545			
Creatinine	0.010	1.010(1.005,1.016)	<0.001***	0.009	1.009(1.003,1.015)	0.004**	0.007	1.007(1.001,1.013)	0.021*	0.004	1.004(0.998,1.011)	0.186
Splenectomy history	0.306	1.358(0.638,2.893)	0.428				1.010	2.745(0.982,7.674)	0.054			

* indicated for *p* < 0.05; ** indicated for *p* < 0.01; *** indicated for *p* < 0.001. OHE, overt hepatic encephalopathy; HR, hazard ratio; CTP, Child-Turcotte-Pugh; MELD, Model for end-stage liver disease; V/S ratio, subcutaneous fat area to visceral fat area ratio; Sarco-myo, combined sarcopenia and myosteatosis.

For 2-year death or liver transplantation, age, CTP score, creatinine, and V/S ratio were significant univariate predictors (Table 4). After incorporating these statistically significant variables into the multivariate regression model, the results demonstrated that age, CTP score, and V/S ratio were risk factors for death or liver transplantation. The patients with a high V/S ratio exhibited a 1.55-fold risk of death or liver transplantation (HR 1.547, 95% CI 1.199–1.997, *p* = 0.001).

## 4 Discussion

In this study, age, diabetes, V/S ratio, creatinine, and combined sarcopenia and myosteatosis were predictors of 1-year OHE after TIPS. Age, V/S ratio, and the CTP score were risk factors for postoperative death or liver transplantation. In addition, diabetes showed a strong association with VFA.

Metabolic disorders driven by VAT lead to lipid metabolism alterations and chronic inflammation, which may increase the susceptibility to post-operation complications. The association between visceral fat accumulation and HE in cirrhotic patients after TIPS may be mediated through multiple pathways. VAT is metabolically active, releasing pro-inflammatory signaling molecules such as TNF-*α* and IL-6, as well as adipokines, which exacerbate systemic inflammation (26). Chronic inflammation could impair the integrity of the intestinal barrier, promote dysbiosis, and increase ammonia production by enhancing bacterial urease activity (27, 28). Excessive VAT accumulation dysregulated glucose metabolism, boosting the pathogenesis of type 2 diabetes, which is consistent with findings in this research (29). VAT secretes abundant free fatty acids and cytokines in the portal vein and transfers them to the liver, which alters liver metabolism and enhances insulin resistance (29). Moreover, insulin resistance induced by VAT will further aggravate the increase in serum ammonia, and insulin resistance affects the metabolism of glutamine, a critical ammonia detoxification pathway (30, 31). Mechanically, a large amount of VAT could also increase intra-abdominal pressure, exacerbating portal hypertension and portal venous system shunt (32). The metabolic dysfunction, systemic inflammation, and elevated blood ammonia caused by VAT interact and jointly lead to the occurrence of HE.

It was suggested that subcutaneous fat had a protective effect and was beneficial to glucose tolerance and muscle metabolism (33). A published study reported that female patients with reduced SFA were more susceptible to HE after TIPS procedures (34). Another clinical study involving 35 cirrhotic patients showed similar results that patients with maintained SFA had decreased frequency of post-TIPS HE episodes (22). VFA or SFA only reflects fat accumulation in a single area and cannot comprehensively assess overall fat distribution. The V/S ratio accounts for the simultaneous proportions of VFA and SFA, which can better reflect abnormal fat distribution and a metabolic disorder state. The higher V/S ratio reflects the severe degree of central obesity, accompanied by worse metabolic disorders, including insulin resistance, chronic inflammation, and altered adipokine profiles. A previous study found that the V/S ratio correlated with the risk of nonalcoholic fatty liver disease and hepatic fibrosis (35). At present, more and more studies have shown that the V/S ratio can predict the prognosis of various diseases. In Crohn’s disease, a higher V/S ratio independently predicted disease severity (36). Similarly, one study demonstrated that the SFA/VFA ratio was better than BMI or waist circumference at identifying individuals at risk for cardiovascular events (37). Recently, a study by Elhakim et al. reported that the V/S ratio was not associated with 90-day mortality after TIPS, a finding that appears to contrast with the present study (20). This discrepancy may be explained by differences in the study endpoints. The V/S ratio is often linked to metabolic syndrome and chronic inflammation, and its detrimental effects are thought to manifest over a longer timeframe, primarily influencing medium- or long-term survival. In the current analysis, the V/S ratio was found to be independently associated with 2-year death or liver transplant, suggesting that it may serve as a marker of chronic disease burden that impacts longer-term prognosis.

Di Cola S. et al. found a high prevalence of myosteatosis among patients with cirrhosis (69.5%), which was consistent with this study (63.2%) (24). The impact of combined myosteatosis and sarcopenia on prognosis may be greater than that of sarcopenia alone in cirrhotic patients, who exhibited significantly higher cumulative 1-year mortality and hospitalization rates compared to the group without muscle alterations (24). In this study, combined myosteatosis with sarcopenia was a predictor of 1-year HE after TIPS. The progression of myosteatosis is associated with reduced SMI and elevated visceral fat index (VFI) (38). Skeletal muscle is the main extrahepatic site for ammonia detoxification through glutamine synthesis. Sarcopenia reduces the ability of glutamine synthase and impairs the clearance of ammonia (39). The portal system shunt induced by TIPS exacerbates hyperammonemia by bypassing liver detoxification. Chronic hyperammonemia can cause mitochondrial dysfunction, decrease lipid oxidation, and subsequently lead to myosteatosis (40). This process may disrupt the normal physiological functions of the muscles (41). Myosteatosis is often associated with insulin resistance and lipid-induced inflammation, which may exacerbate metabolic disorders and inflammatory responses (41).

Traditional prognostic models, such as the CTP and MELD scores, primarily focused on hepatic function and mortality but did not consider metabolic and inflammatory factors associated with post-TIPS complications. The CTP score mainly reflects liver function and is used to assess the severity of liver cirrhosis. In addition, ascites and HE are influenced by subjective factors and treatment, which are difficult to standardize (12, 42). The MELD score incorporates bilirubin, INR, and creatinine, and was designed to assess the risk of death. In some cases, the predictive value of MELD may decrease. Infection can increase bilirubin levels, and creatinine levels are affected by diuretics (43). In contrast, the V/S ratio reflects the influence of inflammation and metabolism on postoperative outcomes after TIPS, providing a biomarker for preoperative risk screening. V/S ratio is a CT-based indicator, which is objective and workable. V/S ratio is an effective tool for predicting OHE and TFS, bridging gaps in current prognostic tools by incorporating adipose tissue into post-TIPS prediction.

Research on prognostic nutritional indicators in liver cirrhosis remains limited, particularly those after TIPS procedures. Post-TIPS complications, such as hepatic encephalopathy, are challenging clinical problems that require attention and solutions, making this study clinically valuable. Nanjing Drum Tower Hospital, as one of the largest TIPS centers in Eastern China, ranks among the top in the number of completed TIPS surgeries and has established a relatively comprehensive database on liver cirrhosis in the Chinese population. To our knowledge, this is the first investigation to examine the predictive capacity of fat distribution, specifically the V/S ratio, in predicting post-TIPS outcomes in cirrhotic patients. V/S ratio is a novel, imaging-based biomarker that can serve as a preoperative risk factor and be integrated into preoperative screening protocols. In clinical applications, professionals can screen high-risk patients in advance and strengthen postoperative management, such as improving HE monitor. In addition, for these high-risk patients, preoperative lifestyle modifications can be guided by dietitians, including personalized dietary management, exercise plans, and glycemic control, to reduce visceral adiposity, mitigate metabolic risks, and enhance recovery in this population. Moreover, body composition was measured using CT imaging, which provides precise quantification of fat and muscle areas. This investigation not only identified the V/S ratio as a reliable prognostic marker for post-TIPS but also offered the optimal predictive cutoff value.

This research has some limitations. Previous HE history was self-reported by patients and their families, and the potential subjectivity in reporting HE episodes was a limitation of the study. Although the analysis was conducted on a prospectively maintained database with standardized data collection protocols, our study was a retrospective design. The number of patients with TIPS indications was unevenly distributed among the groups. The number of patients with refractory ascites was much smaller than that with gastrointestinal bleeding, making it difficult to conduct a stratified analysis.

Furthermore, changes in visceral or subcutaneous adiposity before and after TIPS were not assessed. Inflammatory markers (TNF-*α*, IL-6) and insulin resistance parameters, such as HOMA-IR, were not measured in this study. Future studies could integrate these data to verify the inflammatory and metabolic pathways linking fat distribution to TIPS complications.

## 5 Conclusion

In summary, the high V/S ratio was associated with a 1.75-times 1-year OHE risk and a 1.55-fold risk of death/liver transplantation compared to patients with the low V/S ratio. Preoperative assessment of VFA and SFA is not only beneficial for monitoring the nutritional status of patients with liver cirrhosis but also helpful for predicting HE after TIPS and long-term TFS. Early intervention for patients with a high V/S ratio improves the clinical prognosis of TIPS and further enhances patients’ overall quality of life.

## Data Availability

The datasets presented in this article are not readily available due to privacy and ethical restrictions. Requests to access the datasets should be directed to the corresponding author.

## Supplementary material

SUPPLEMENTARY FIGURE 1 Flow chart of the research.

## Glossary

TIPS: Transjugular Intrahepatic Portosystemic Shunt
HE: Hepatic Encephalopathy
OHE: Overt Hepatic Encephalopathy
CTP: Child-Turcotte-Pugh
MELD: Model For End-Stage Liver Disease
VAT: Visceral Adipose Tissue
BMI: Body Mass Index
SMI: Skeletal Muscle Index
Sarco-myo: Combined Sarcopenia and myoteaotosis
SFA: Subcutaneous Fat Area
VFA: Visceral Fat Area
V/S Ratio: Subcutaneous Fat Area To Visceral Fat Area Ratio
HU: Hounsfield Unit
SD: Standard Deviation
ROC: Receiver Operating Characteristic
AUROC: Area Under Receiver Operating Characteristic Curves
Cl: Confidence Intervals
HR: Hazard Ratio
ALT: Alanine Aminotransferase
AST: Aspartate Aminotransferase
TBIL: Total Bilirubin
WBC: White Blood Cell
PT: Prothrombin Time
INR: International Normalized Ratio
RAP: Right Atrial Pressure
IVCP: Inferior Vena Cava Pressure
PVP: Portal Venous Pressure
