# Supplementary figures and images for: Visceral to subcutaneous fat area ratio as a novel prognostic biomarker in cirrhosis patients undergoing TIPS: a retrospective study
Source: Front Med (Lausanne). 2026 Mar 19;13:1769708. doi: 10.3389/fmed.2026.1769708 (PMC13044108; doi:10.3389/fmed.2026.1769708)

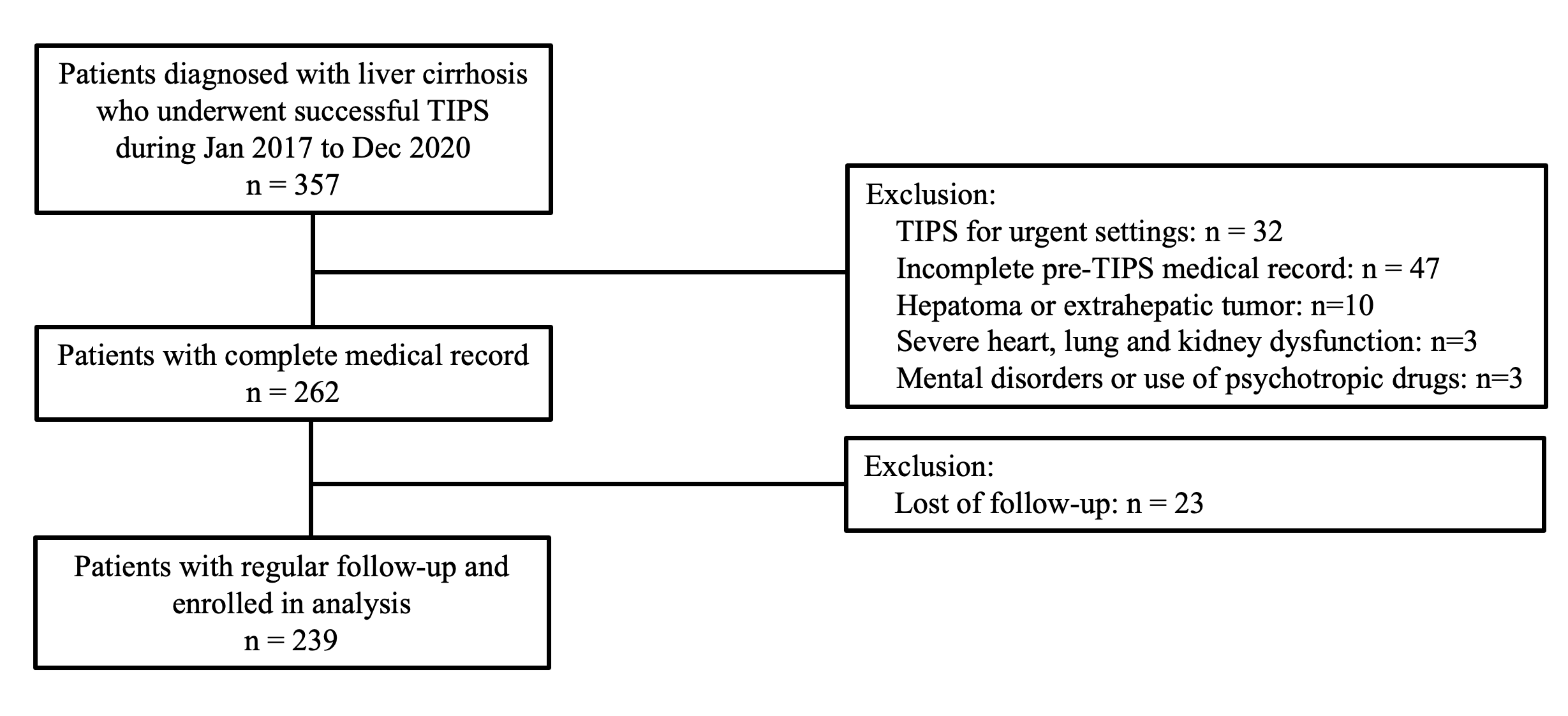

Supplement: SUPPLEMENTARY FIGURE 1 — Flow chart of the research. [file Image_1.TIFF]
